# Toxic Impacts of Cypermethrin on Behavior and Histology of Certain Tissues of Albino Rats

**DOI:** 10.4103/0971-6580.72679

**Published:** 2010

**Authors:** K. K. Grewal, G. S. Sandhu, Ranjit Kaur, R. S. Brar, H. S. Sandhu

**Affiliations:** Department of Zoology, S.G.N. Khalsa College, Sri Ganganagar, Rajasthan, India; 1 Department of Veterinary Pathology, GADVASU, Ludhiana, India; 2 Department of Veterinary Pharmacology and Toxicology, GADVASU, Ludhiana, India

**Keywords:** Albino rats, cypermethrin, histopathology, insecticides, pyrethroids

## Abstract

In the present investigation, the behavioral, morphological, and histopathological effects of cypermethrin, a widely used synthetic pyrethroid insecticide, was ascertained in male and female albino rats (*Rattus norvegicus*). Cypermethrin administered at repeated oral doses of 5 and 20 mg/kg/day for 30 days produced varying degree of mild to moderate toxic symptoms and behavioral changes in both male and female rats. The lower dose produced very mild toxicosis characterized by intermittent diarrhea, decreased feed intake, and thick eye discharge, whereas higher dose displayed mild to moderate toxicosis with diarrhea, decreased feed intake, loss of body weight, dyspnoea, ataxia, eye discharge, and salivation. Two female and one male albino rats died between 23 to 28 days after displaying signs of incoordination and tremors. Repeated oral doses of cypermethrin for 30 days enhanced the relative weight of liver and heart, but significantly decreased that of brain, kidneys, and testes. Microscopically, cypermethrin produced neuronal degeneration and increase in glial cells in brain, and disorganization of hepatic laminae, increase in sinusoid, and necrosis of hepatocytes in liver. Section of kidney displayed hemorrhage and sloughing off renal epithelial cell in the convoluted tubules, shrinkage of glomeruli, and necrosis of renal tubules. Repeated administration of cypermethrin also produced hemorrhages within myocardium, disruption of branching structure, and loss of striation of cardiac tissue; thickening of alveolar septa in lungs, partial to extensive loss of various stages of spermatogenesis in testes, and loss of follicular cells and oocytes in ovaries. The study suggested that repeated oral exposure of cypermethrin has considerable harmful effects on body organs in *R. norvegicus*.

## INTRODUCTION

The modern use of insecticides has substantially improved the economic and social well being of the inhabitants of developing world by increased food production and by the effective control of public health vector-borne diseases. At the same time, public concern over the amounts of insecticides that are being applied to the land and their possible adverse effect on human and animal health, and on the environment has risen sharply. Man has become victim of his own advances with upsurge of many unexplained ailments. Large-scale population declines of many species of birds, mainly fish-eating and bird-eating species, have been ascribed to the exposure to insecticides through food chains and upward biomagnifications of residues. Blood disorders (anemia, defective blood coagulation), brain and nerve damage, paralysis, jaundice and hepatic fibrosis, allergenic sensitization, emphysema, asthma, kidney problems, cancer, genetic disorders, birth defects, miscarriage, impotence, and infertility or sterility are often associated with low residual level of pesticides.[1,2] In animals, interrupted estrous cycle, decreased weight gain, decreased appetite, decreased milk production, or reproductive problems can be correlated with continues low-level exposure of pesticides.[3]

In a search to find new insecticides of low mammalian toxicity, less insect resistance, and low persistence, a number of Groups of insecticides have been synthesized, and synthetic pyrethroids is the result of one of such attempt. Problems of mutagenicity, teratogenicity, or carcinogenicity with pyrethroids are rare, as they do not appear to pose serious residue problems in the food and food products.[4] However, recent literature on synthetic pyrethroids have suggested that some newly introduced members, especially those belonging to α-cyano pyrethroid subGroup, are not free from adverse effects and can pose serious health problems if not handled properly. Like other synthetic pyrethroids, the use of cypermethrin, a fourth generation synthetic pyrethroid, is increasing in agriculture as the pests are becoming resistance to organophosphorus and organochlorine insecticides. Like other insecticides, the wide-spread use of cypermethrin has been associated with adverse effects on nontarget species. Lessengu[5] reported cypermethrin poisoning in human beings due to inhalation of the insecticide when it was used as a termiticide and entered the air conditioning. Kolf-Clauw and Poletti[6] reported incidence of cypermethrin toxicosis in cats and dogs, where it produced nervous and digestive symptoms. Keeping in view its wide application in agriculture and its associated harmful effects, the present study was designed to evaluate the behavioral, morphological, and histopathological effects of cypermethrin by repeated oral administration in *Rattus novegicus*.

## MATERIALS AND METHODS

A total of 70 albino rats (35 males and 35 females) weighing between 150 and 200 g were produced commercially from M/s The Engineer’s Scientific Zoological Farm, Amritsar. The rats were maintained in standard polypropylene rat cages with stainless steel top grills under laboratory conditions (Temperature, 20 – 25°C). The rats were divided into three Groups viz. I, II, and III. The Groups I and II comprising of 30 animals (15 males and 15 females) each were further subdivided into three sub Groups viz. Ia, Ib, Ic and IIa, IIb, IIc, each having 10 animals (five males and five females). Cypermethrin was administered to all animals of group I at dose rate of 5 mg/kg/day and those of group II at 20 mg/kg/day. The Group III comprising of 10 animals (five males and five females) served as control and did not receive any insecticide. The requisite amount of cypermethrin was suspended in Arachis oil and administered orally with the help of rat stomach pipe daily between 9.00 and 10.00 AM. All animals were provided with normal feed and water *ad libitum* during the study period.

Cypermethrin-exposed animals were observed closely during the study period for appearance of behavioral changes and toxic symptoms. The nature, degree, and time of occurrence of toxic symptoms were carefully recorded. Body weights of all animals were recorded at an interval of 10 days. The animals of three subGroups of Group I (Ia, Ib, Ic) and Group II (IIa, IIb, IIc) were sacrificed by cervical dislocation on days 10, 20, and 30, respectively. Animals of Group III (control Group) were sacrificed on the day 30 after completion of the study. Necropsy was conducted on all animals to observe gross morphological changes. Various tissues viz. brain, liver, kidney, heart lungs, and testes/ovaries were dissected out, cleaned with physiological saline solution (0.9% NaCl) to make them blood free, and blotted on a filter paper. Live weight of animals (before necropsy) and weight of various tissues after necropsy were taken on a single pan electronic balance. Tissues were put in 10% buffered formalin for subsequent processing and histopathological studies. The formalin-fixed tissues were thoroughly washed in running tap water, dehydrated in ascending grades of alcohol, cleared in benzene, and embedded in paraffin at 58°C. 5 *µ*-thick sections from paraffin-embedded tissues were stained by hematoxylin and eosin (H and E) method.[7] Slides were examined by (AHBT) Research Photomicrographic Microscope System of Olympus Corporation, USA.

## RESULTS AND DISCUSSIONS

Cypermethrin dose at the rate of 5 and 20 mg/kg/day produced varying degree of mild to moderate toxic symptoms and behavioral changes in albino rats. At the lower dose of 5 mg/kg/day, cypermethrin produced mild to moderate toxicosis characterized by intermittent diarrhea, decreased feed intake, and thick eye discharge. One female rat died on the 28^th^ day of the insecticide treatment. Animals exposed to higher dose (20 mg/kg/day) produced mild to moderate toxicity characterized by diarrhea, decreased feed intake, loss of body weight, dyspnoea, ataxia eye discharge, and salivation. Two female and one male albino rats died in between 23 and 28 days of the insecticide treatment, after displaying signs of incoordination and tremors. The study showed that nature and intensity of toxic symptoms produced by cypermethrin are dose and time dependent. The observed signs were somewhat similar to those reported by other workers following repeated administration of deltamethrin and cypermethrin in rodents.[8] Neuschl *et al*.[9] studied the toxic effect of super cypermethrin, a cypermethrin analogue, in pheasants after repeated oral exposure and reported clinical signs of mild diarrhea. Ataxia and behavioral changes produced by acute and subacute doses of pyrethroids have been related to sciatic nerve degeneration[10] and alterations in regional brain polyamine levels.[11]

**Figure 1:**
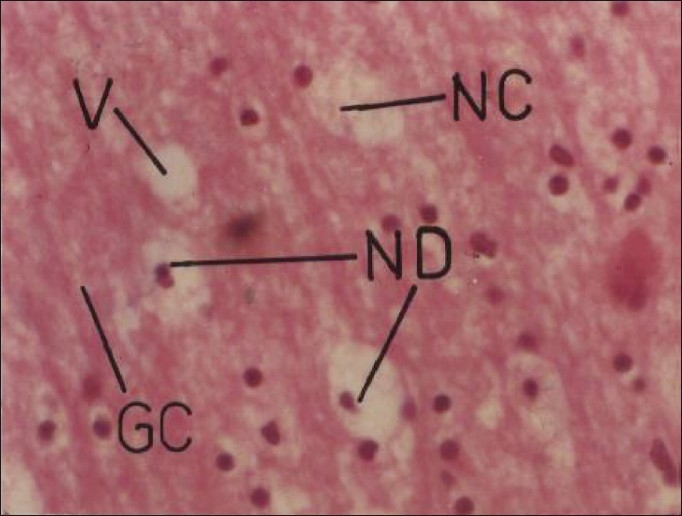
Photomicrographs of a section of cerebrum of rat showing necrosis and vacuolization with neuronal degeneration and marked increase in glial cells. H and E, ×200 (Cypermethrin, 20 mg/kg/day, 30 day) GC = glial cells; NC = necrosis; ND = neuronal degeneration; V = vacuole

**Figure 2:**
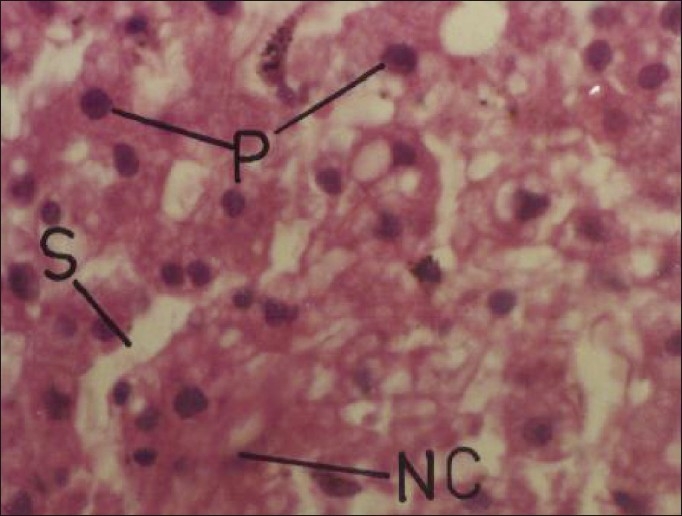
Photomicrographs of a section of liver of rat showing necrosis of hepatic cells with pyknotic nuclei, disorganization of hepatic laminae, and dilation of sinusoids. H and E, ×200 (Cypermethrin, 20 mg/kg/day, 30 day) NC = necrosis; P = pyknotic nuclei; S = sinusoid(s)

Cypermethrin at repeated oral doses of 5 and 20 mg/kg/day did not produce any apparent morphological changes in the brain. However, the relative weight of brain significantly decreased to the extent of 22.95% and 15.09% in male and female rats, respectively. Cypermethrin at daily oral dose of 5 mg/kg produced mild neuronal degeneration and vacuolization in the cerebral hemisphere in rats on 30^th^ day. Higher dose (20 mg/kg/day) of insecticide produced neuronal degeneration and vacuolization on 20^th^ day and neuronal degeneration and necrosis with marked increase in glial cell on 30^th^ day [Figure 1]. These findings are in agreement with Gupta[8] who reported extensive neuronal damage and glial cell proliferation with repeated oral doses of cypermethrin. Many pesticide agents are reported to cause variable changes in brain on repeated exposure,[12] which have been related to hypoxia, hypoglycemia, and/or damage to cell ion homeostasis.[13] Organometry studies on the liver also suggested significant increase in the relative weight to the extent of 37.82% (females) and 37.89% (males) after 30 days of insecticide treatment. The increased relative weight of liver was probably due to the functional hypertrophy of the smooth endoplasmic reticulum and increased drug metabolizing multienzyme complex as suggested by Zimmerman[14] and Ayub-Shah *et al*.[15] Microscopically, lower dose of cypermethrin produced mild disorganization of hepatic laminae and higher dose produced necrosis of hepatic cells, with pyknotic nuclei and dilatation of sinusoids with highly disrupted hepatic laminae [Figure 2] in both male and female rats. The observed findings are similar to Biernacki *et al*.[16] who reported variable hepatotoxic effect of cypermethrin in rabbits. Fatty degeneration and necrosis of hepatocytes have been observed following exposure of animals to several heptotoxicants.[17]

**Figure 3:**
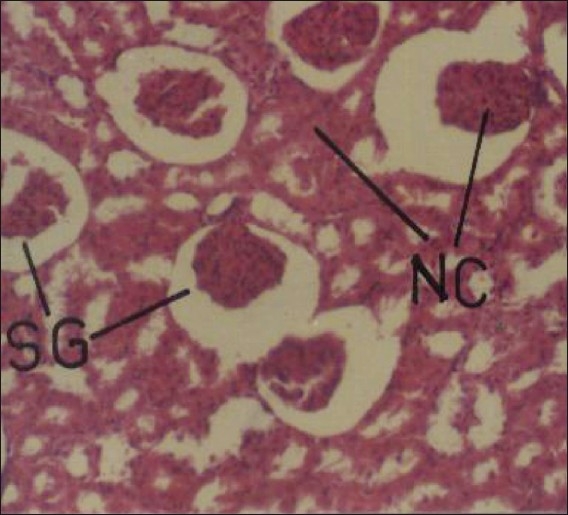
Photomicrographs of a section of kidney of rat showing shrinkage of glomeruli, necrosis and disruption of renal tubules. H and E, ×100 (Cypermethrin, 20 mg/kg/day, 30 day) NC = necrosis; SG = shrinkage of glomeruli

**Figure 4:**
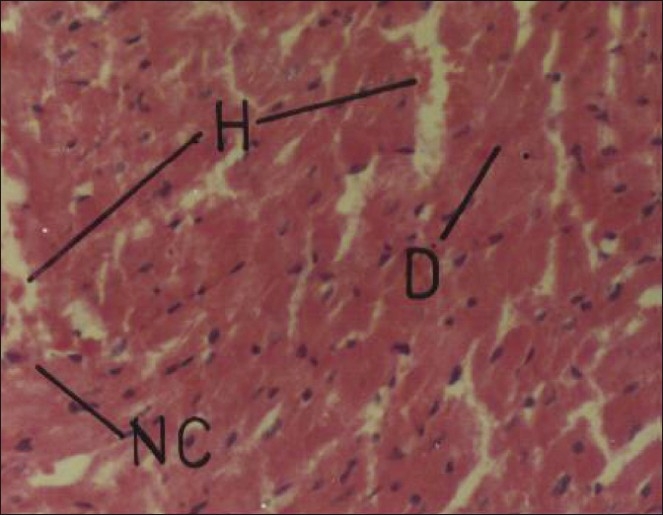
Photomicrographs of a section of heart of rat showing hemorrhage, disorganization of branching structure with loss of striations, and early necrotic changes in myocardium. H and E, ×200 (Cypermethrin, 20 mg/kg/day, 30 day) D = disorganization, H = hemorrhage; NC = necrosis

Cypermethrin at repeated oral doses of 5 and 20 mg/kg/day produced no apparent morphological changes in kidney in the male and female rats. However, relative weight of kidneys significantly reduced to the extent of 27.27% (males) and 17.07% (females). Cypermethrin produced significant alterations in the histoarchitecture of kidney, especially at higher dosage, in both male and female rats. Lower dose produced sloughing off renal tubular epithelial, but no effect of glomeruli. Higher dose produced mild hemorrhage, sloughing off epithelial cell, shrinkage of glomeruli, and necrosis of renal tubules [Figure 3] on 30^th^ day of insecticide treatment. The observed results are in accordance with Majumder *et al*.[18] who reported glomerular and tubular necrosis in broiler chicks treated with oral doses of fenvalerate. Alden and Frith[19] have suggested that any process that interferes with the structural integrity of the glomeruli and renal tubules can cause renotoxic effect. In such cases, leakage of lysosomal enzymes may occur, thereby causing cell necrosis and renal damage. Cypermethrin dose at 5 and 20 mg/kg/day did not produce any gross abnormality in heart, but produced significant increase in its relative weight to the extent of 33.33% (males) and 21.74% (females). Histopathological studies on heart showed that lower dose of cypermethrin has no apparent adverse effect, but higher dose produced hemorrhages, disruption in branching structure with loss of striations, and early necrotic changes in the myocardium [Figure 4]. The changes observed in heart suggested mainly prolonged harmful effect of cypermethrin. Although the cause of necrosis of cardiac tissue in the present study cannot be cited with precision, several factors including ischemia, metabolite toxicities, and/or endocrine disturbances are reported to cause such myocardial defects.[20]

**Figure 5:**
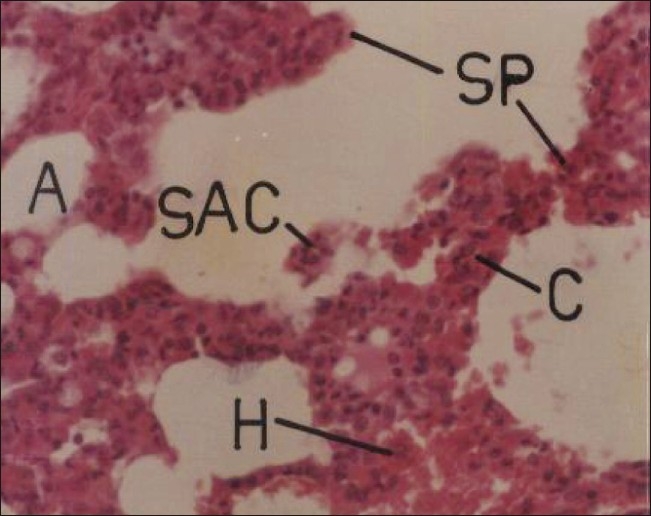
Photomicrographs of a section of lung of rat showing congestion and hemorrhage with thickening of interalveolar septa. Some sloughed-off degenerating alveolar cells are seen lying within lumen of alveoli. H and E, ×200 (Cypermethrin, 20 mg/kg/day, 30 day) A = alveolus/alveoli; C = congestion; H = hemorrhage; SAC = sloughed-off alveolar cells; SP = interalveolar septum/septa

Necropsy conducted on male and female rats intoxicated with repeated oral doses of 5 and 20 mg/kg/day after 30 days treatment neither showed any gross morphological changes nor any significant alteration in the relative weight of lungs. However, photomicrographs of a section of lung [Figure 5] showed dose- and time-dependent histopathological alterations with congestion, hemorrhage, and thickening of interalveolar septa. The observed findings suggested that cypermethrin produced irritant effect on the pulmonary tissue, because proliferation of fibroblasts located in the pulmonary interstitium frequently follows injury and continuous irritation.[21] Excessive fibroblast proliferation and collagen production resulting in fibrosis and thickening of alveolar septa compromise pulmonary elasticity or compliance, and can affect gaseous diffusion.[22]

**Figure 6:**
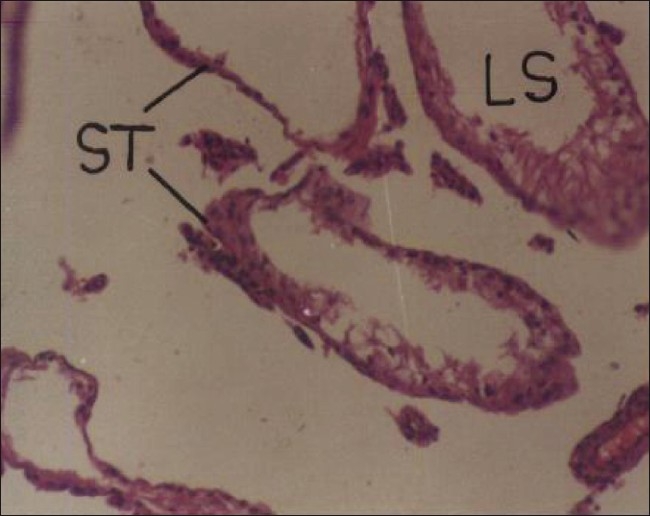
Photomicrographs of a section of testis of rat showing deformed seminiferous tubules with extensive loss of various stages of spermatogenesis. H and E, ×100 (Cypermethrin, 20 mg/kg/day, 30 day) LS = loss of spermatozoa; ST = seminiferous tubules

**Figure 7:**
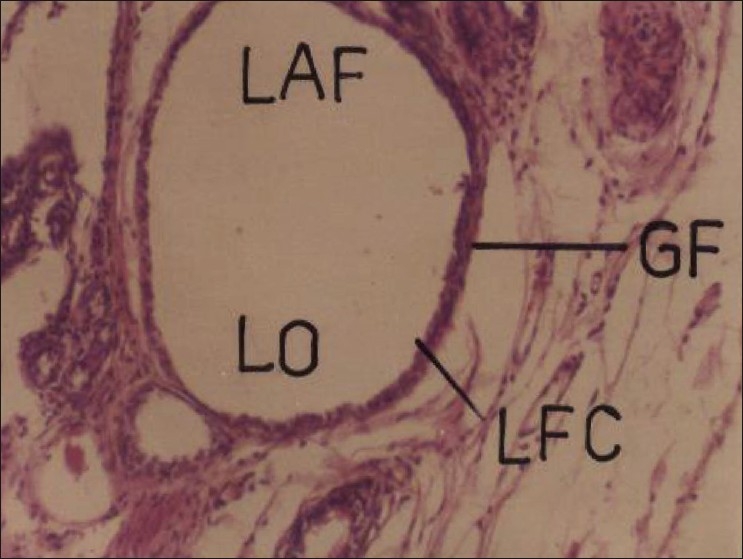
Photomicrographs of a section of ovary of rat showing complete loss of follicular cells, oocyte, and albuminous fluid in the Graafian follicle. H and E, ×200 (Cypermethrin, 20 mg/kg/day, 30 day) GF = Graafian follicle; LAF = loss of albuminous fluid; LFC = loss of follicular cells; LO = loss of oocyte

Repeated oral administration of cypermethrin (5 and 20 mg/kg/day) produced no apparent morphological changes in the testes. Grossly, the size of testes was found smaller in insecticide treated male rats and the organometry studies showed significant reduction (56.12%) in the relative weight of testis in the higher dose Group animals. Histopathological studies on cypermethrin suggested extensive damage to somniferous tubules and alterations in the histoarchitecture of testis. Cypermethrin at higher dose produced partial (20 day) to extensive (30 day) loss of various stages of spermatogenesis [Figure 6]. Similar to the present findings, Cigankova *et al*.[23] observed degeneration and depletion of spermatocytes and spermatids in supermethrin-exposed adult pheasants. Creasy and Foster[24] suggested that necrosis and loss of germ cells are the most frequent manifestations of testicular injury but also are the most difficult to interpret due to the dynamic nature of germ cell development. In female rats, repeated oral administration of cypermethrin at both doses showed significant effect on the histopathology of ovary. Higher dose produced complete loss of follicular cells, oocyte, and albuminous fluid in the Graafian follicle [Figure 7]. Reproductive study is less often studied in the female, because reproduction in the female involves a complex series of interdependent and interrelated steps.[25] Adverse effect of toxicants on the ovary can result from reduced gonadotropin secretion, impairment of follicular growth, or enzymatic interference, resulting in a reduction of sex steroid hormone synthesis.[26]

From the present study, it may be concluded that repeated oral exposure of cypermethrin has considerable harmful effects on body organs in albino rats. The observed neuro- hapato- reno- and pulmono-toxicity are likely to disrupt neuronal, metabolic, excretory, and respiratory processes resulting in serious debilities. Adverse effects of cypermethrin on male and female gonads clearly depicted hazardous effects of the pyrethroid insecticide on reproductive processes, particularly the fertility in both male and female rats.
